# Minimizing Technical Failure of Percutaneous Balloon Compression for Trigeminal Neuralgia Using Neuronavigation

**DOI:** 10.1155/2014/630418

**Published:** 2014-03-09

**Authors:** Miltiadis Georgiopoulos, John Ellul, Elisabeth Chroni, Constantine Constantoyannis

**Affiliations:** ^1^Functional Neurosurgery Unit, Department of Neurosurgery, Faculty of Medicine, University of Patras, 26500 Patras, Greece; ^2^Department of Neurology, Faculty of Medicine, University of Patras, 26500 Patras, Greece

## Abstract

*Objective*. Percutaneous balloon compression (PBC) is an effective and safe management for medically refractory trigeminal neuralgia; however, technical failure to cannulate the foramen ovale (FO) using only fluoroscopy is a significant problem in some cases. In this paper, we suggest the use of intraoperative navigation, in cases of reoperation due to prior technical failure to cannulate the FO under fluoroscopy. *Methods*. A total of 174 patients underwent PBC for TN since 2003. In 9 cases the penetration of the FO was not accomplished. Five of those patients were reoperated on for PBC using navigation from March 2012 to September 2012. Surgical technique: preoperatively, a head Computed Tomography (CT) scan is performed and the acquired images are imported into the navigation system. Intraoperatively, a small reference frame is strapped firmly to the patient's forehead, the CT images are registered, and cannulation is performed under the guidance of the navigation system. *Results*. In all patients, the operation overall was completed successfully. Moreover, all patients reported complete pain relief immediately postoperatively and no complications were recorded overall. *Conclusions*. We suggest the use of neuronavigation in cases of technical failure of PBC. That technique involves technology with significant advantages helping the successful cannulation of the FO and seems more efficient and safer.

## 1. Introduction

Trigeminal neuralgia (TN), the most common craniofacial pain syndrome with an annual incidence of 3–5/100.000, can be a torturing condition devastating the patient's quality of life [1, 2]. Half of the patients suffering from TN need an operation eventually, because of relapse or severe side effects of the drugs [2, 3].

Currently, the most popular therapeutic interventions for medically refractory TN include microvascular decompression, stereotactic radiosurgery, and percutaneous procedures, that is, percutaneous balloon compression (PBC), radiofrequency (RF) rhizotomy, and glycerol rhizotomy [2, 4–7]. Microvascular decompression provides the most long-lasting relief among the above techniques with the lowest recurrence rate [1, 4, 8, 9]. PBC is a reliable, effective, and safe technique [8–10]. Along with RF rhizotomy, it is one of the most effective ablative techniques, characterized also by a relatively low morbidity (16.1%) [8–10].

Typically, PBC is performed under the guidance of fluoroscopy (lateral, anteroposterior, and oblique submental views), but visualization of the FO can be inadequate sometimes and the exposure to radiation during fluoroscopy could be significant for the surgeon [11]. Also, the size of the foramen ovale (FO) is relatively small (6.5 × 3 mm) in contrast to the thick cannula used in PBC (14 gauges) [12, 13]. Moreover, the percutaneous approach of the FO is sometimes difficult, due to the presence of anatomical variations, such as ossified pterygospinous or pterygoalar ligaments or other intraforaminal bony ridges [4, 14, 15]. As a result, failure to cannulate the FO in PBC has been documented in up to 8% of the cases [16]. Also, multiple attempts to cannulate the FO might increase the risk of complications, such as vascular injuries or abducens nerve and other cranial nerve deficits [9, 10, 17].

For the above reasons, intraoperative navigation, which has been used successfully in various neurosurgical operations, could allow for an accurate targeting of the FO, in cases of prior failure to cannulate it using fluoroscopy [12, 18–22]. The advantages of intraoperative navigation systems include three-dimensional planning (preoperative and intraoperative), real time instrument guidance, and accurate localization of intracranial targets [12].

In this paper, we suggest a treatment alternative: the use of intraoperative navigation for the guidance of PBC for TN, in cases of reoperation after prior technical failure to cannulate the FO under fluoroscopy.

## 2. Materials and Methods

### 2.1. Patients

A total of 174 patients underwent PBC for TN since 2003. In 9 cases the penetration of the FO was not accomplished and a narrow FO was suspected as a reason. Five of those patients, suffering from medically refractory primary TN, were reoperated on for PBC using navigation from March 2012 to September 2012. Four patients denied reoperation in spite of the persistence of pain.

Three patients were male and 2 female; their age ranged from 50 to 77 years and their disease duration from 2 to 11 years. Left trigeminal nerve was affected in 4 patients; the distribution of all branches was involved in 2, of the maxillary nerve in 2, and of V_1_-V_2_ in one. All procedures were performed by a single neurosurgeon. The research protocol has been approved by the Scientific and Ethics Committee of the University Hospital of Patras, Patras, Greece, and of the Faculty of Medicine of the University of Patras, Patras, Greece; and informed consent has been obtained from each patient.

### 2.2. Surgical Technique

**Figure 1:**
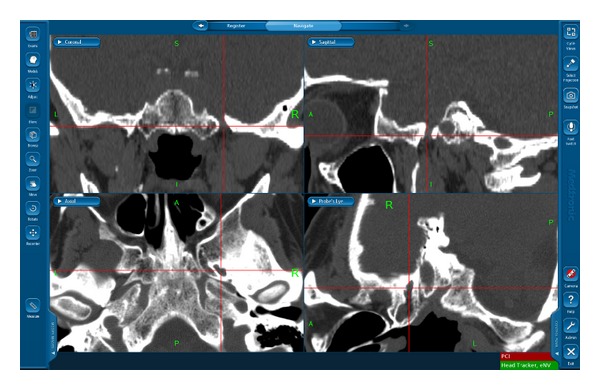
The left FO is identified in coronal, sagittal, axial, and probe's eye views of the navigation system (StealthStation S7, Medtronic Inc., Minneapolis, MN, USA).

A head CT scan (bone windows) is performed at 1.5 mm intervals, without contrast agent, one day before the operation. The acquired images are imported into the planning and navigation system (StealthStation S7, Medtronic Inc., Minneapolis, MN, USA); coronal, sagittal, probe's eye, and 3D reconstructions are created, the FO is identified, and the ideal trajectory is designed (Figure 1). During the operation, the navigation system is combined with a small reference frame (Head Tracker Frame, Medtronic Inc., Minneapolis, MN, USA) and an adapter (SureTrak II, Medtronic Inc., Minneapolis, MN, USA) to make the cannula used in PBC identifiable by the navigation system.

In the operating room, after the induction of general anesthesia, the head of the patient is positioned in slight extension on a horseshoe headrest. The Head Tracker Frame is strapped firmly to the patient's forehead. The CT images are registered with the Passive Planar Blunt Probe (Medtronic Inc., Minneapolis, MN, USA) by placement of the probe on the Head Tracker Frame and specific facial features (e.g., nasal tip, nasion), indicated by the Synergy Cranial 2.0 software. The cannula (a rigid Tuohy type 14-gauge needle) is tracked by the navigation system with the SureTrak II adapter.

**Figure 2:**
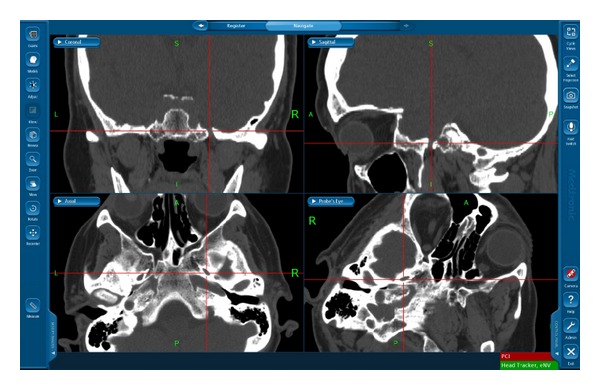
Axial, coronal, sagittal, and probe's eye views on the same window of the navigation system used to cannulate the FO. The left FO was relatively narrower compared with the contralateral one, which might explain the initial technical failure of the PBC under fluoroscopy.

Cannulation is performed under the guidance of the navigation system using 4 views on the same window of the Synergy Cranial 2.0 software: axial, coronal, sagittal, and probe's eye views (Figure 2). In the end, fluoroscopy can be used only to confirm the penetration into the FO, inflation of the balloon into the Meckel's cave and the C-arm fluoroscopic display can be transferred on the navigation screen (instead of another view).

After the successful cannulation and the fluoroscopic confirmation, the procedure is completed in the usual fashion, as it has been described in previous papers [17]. After 3 min of constant pressure application, the balloon is deflated and withdrawn together with the cannula. Mechanical compression is then applied for 5 min on the cannula's point of insertion on the cheek, to prevent hematoma formation.

The method is presented in brief in Table 1.

## 3. Results and Discussion

### 3.1. Results

In all patients, the cannulation of the FO was accomplished with one attempt and the operation overall was completed successfully and conveniently. Furthermore, in 3 cases the FO of the target side was clearly narrower compared with the contralateral side of the skull base, as shown by the preoperative bone CT scan (Figure 2). The appropriate location of the inflated balloon was confirmed by fluoroscopy in the end. All patients reported complete pain relief immediately postoperatively. The patients were discharged on the first postoperative day, because it is routine practice in our center to observe their vital functions, consciousness level, and pain alleviation for one day.

No complications were recorded overall, except for the expected mild hypesthesia at the trigeminal distribution, which did not involve the cornea. On average, an additional time of 15 minutes (range: 12–17) was needed to prepare the navigation system for the real time guidance. All patients remain free of pain in the follow-up (23–30 months).

## 4. Discussion

Various methods have been described for the cannulation of the FO using CT and navigation systems, in order to promote successful insertion with the least possible attempts and avoid complications, as well [12, 18, 19, 23–25]. However, we have not encountered any other paper analyzing the application of a navigation system, without impractical frames or intraoperative imaging systems, specifically for PBC and TN, after technical failure to cannulate the FO under fluoroscopy.

The technique analyzed in this report does not involve cumbersome frames for navigation's reference or stereotaxy, head clamp fixation, or fiducials. Bale et al. have described the use of an immobilization device with two mechanical arms in combination with an individualized dental mold and an aiming device, too [12]. Except for the patient's comfort and avoidance of postoperative pain because of head clamp fixation or fiducials, a small practical reference frame, like the Head Tracker Frame, can provide an ease in the maneuvers during the operation.

The CT images used by the navigation systems offer a superior resolution of the skull base osseous structures to the classical fluoroscopic images, while the X-ray exposure from fluoroscopy is reduced significantly. Fluoroscopy during the protocol proposed in this paper was limited to one exposure, making the procedure safer for the surgeon. The simultaneous display of different views (axial, coronal, sagittal, and probe's eye) or 3D reconstructed images is much more visuospatially informative, especially in cases with anatomical variations [24].

Intraoperative imaging systems have been used in some studies [12, 18, 24]. Those systems are not widely available due to their cost. Besides, they may not provide an actual additional benefit. If cannulation is completed under navigation guidance using preoperative imaging as described above, it can be confirmed by C-arm fluoroscopy using a common device. If cannulation cannot be achieved even with navigation, sequential CT guidance could be used according to Lin et al. [24].

RF rhizotomy has been applied in almost all studies describing the use of navigation systems in percutaneous procedures for TN [12, 19, 23–25]. According to reviews, PBC is equally effective in the long term and has a lower complication rate (16.1%) than RF rhizotomy (29.2%), especially concerning anesthesia dolorosa and keratitis [8–10]. PBC has been used only in one study using navigation, in which an intraoperative imaging system was used, as well [18].

## 5. Conclusions

Surgical management of unsuccessful PBC owing to technical reasons remains controversial, since neurosurgeons may offer another option, such as glycerol injections or RF rhizotomy. In this report we suggest the use of navigation for the guidance of the cannula during PBC, in cases of prior failure to penetrate into the FO under fluoroscopy. That technique involves technology with significant advantages helping the successful cannulation of the FO and seems more convenient, more efficient, and safer. Nonetheless, the latter has to be confirmed by clinical studies of sound methodology, for example, prospective cohort studies or randomized controlled studies.

## Figures and Tables

**Table 1:** Surgical technique in steps.

Preoperatively	
(1) Head CT scan (bone windows) is performed	
(2) Acquired CT images are imported into the navigation system	

Inside the operating room	
(3) The Head Tracker Frame is strapped firmly to the patient's forehead	
(4) The CT images are registered with the Passive Planar Blunt Probe	
(6) Cannulation is performed under the guidance of the navigation system	
(7) Cannulation is confirmed by C-arm fluoroscopy	
(8) The Fogarty catheter is slowly inflated at the entrance of Meckel's cave and kept in place for 3 minutes	
(9) Balloon is deflated and withdrawn together with the cannula	
